# Hypoxia-inducible factor-1alpha protects the liver against ischemia-reperfusion injury by regulating the A2B adenosine receptor

**DOI:** 10.1080/21655979.2021.1953217

**Published:** 2021-07-21

**Authors:** Xingjian Zhang, Peng Du, Kaifeng Luo, Yong Li, Zhongzhong Liu, Wei Wang, Cheng Zeng, Qifa Ye, Qi Xiao

**Affiliations:** a Department of General Surgery, The First Affiliated Hospital of Nanchang University, Nanchang, Jiangxi, China; b Institute of Hepatobiliary Diseases, Transplant Center, Hubei Key Laboratory of Medical Technology on Transplantation, Zhongnan Hospital, Wuhan University, Wuhan, Hubei, China

**Keywords:** Ischemia-reperfusion injury, liver, A2B adenosine receptor, hypoxia inducible factor-1α

## Abstract

Hepatic ischemia-reperfusion injury (IRI) is an inevitable complication associated with liver surgical procedures, and its pathological process remains elusive. Therefore, the present study investigated the role and mechanism of hypoxia-inducible factor-1alpha (HIF-1α) in hepatic IRI. Here, we constructed rat models with hepatic IRI and BRL-3A cell models with hypoxia/reoxygenation (H/R) insult. The extent of liver injury was assayed by measuring serum ALT/AST levels and performing H&E staining; the levels of SOD, MDA, MPO, IL-6 and TNF-α were determined using commercial kits; apoptosis was detected using the TUNEL assay and flow cytometry; and the expression of HIF-1α/A2BAR signaling-related molecules and apoptosis-associated indicators was detected using Western blotting or qRT-PCR. The expression level of HIF-1α was significantly upregulated in the liver of rats subjected to IRI, as well as in BRL-3A cells treated with H/R. HIF-1α overexpression exerted a protective effect on hepatic IRI or H/R insult by reducing serum aminotransferase levels and hepatic necrosis, inhibiting inflammation and apoptosis of hepatocytes, and alleviating oxidative stress. In contrast, inhibition of HIF-1α expression exacerbated hepatic injury induced by IR or H/R. Mechanistically, the expression level of A2BAR was markedly increased during hepatic IRI or H/R insult. Moreover, A2BAR expression increased with HIF-1α upregulation and decreased with HIF-1α downregulation. Importantly, inhibition of A2BAR signaling abolished HIF-1α overexpression-mediated hepatoprotection. Taken together, HIF-1α exerts protective effects on hepatic IRI by attenuating liver necrosis, the inflammatory response, oxidative stress and apoptosis, and its mechanism may be related to the upregulation of A2BAR signaling.

## GRAPHICAL ABSTRACT

**Figure:**
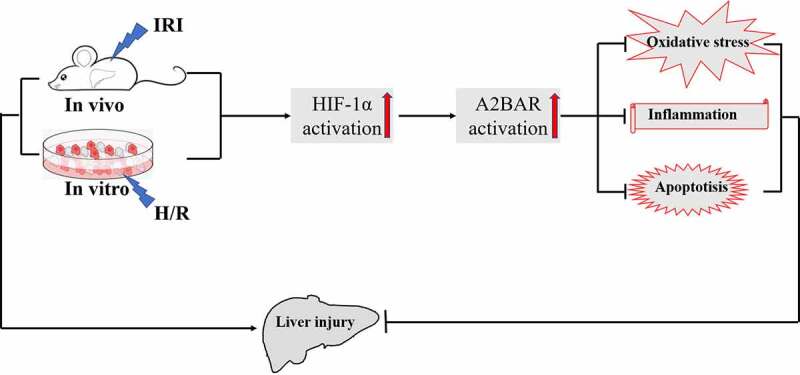

## Introduction

Ischemia-reperfusion injury (IRI) is an inevitable complication associated with hepatic trauma surgery, hepatic resection and transplantation, which is a two-stage pathophysiological phenomenon characterized by an initial restriction of blood flow to an area or organ causing damage and subsequent restoration of blood flow to aggravate the damage [1,2]. Hepatic IRI during liver resection or liver transplantation potentially contributes to elevated liver enzyme levels, biliary strictures, clinical dysfunction, and even liver failure, and is a major cause of morbidity and mortality following hepatic surgery [3,4]. Based on accumulating evidence, several factors play a role in the pathogenesis of IRI, such as cell apoptosis, necrosis, oxidative stress, and inflammation [5]. However, the precise mechanisms responsible for hepatic IRI remain unclear, and efficient interventions to minimize this injury are still limited in number. Therefore, investigations of the molecular pathophysiological mechanisms underlying hepatic IRI are essential to develop new targets and strategies for further treatment.

Hypoxia-inducible factor-1 (HIF-1), which is composed of an oxygen-destructible HIF-α subunit and an oxygen-indestructible HIF-1β subunit, principally mediates the transcriptional response of mammalian cells to hypoxia [6]. Under normoxic conditions, HIF-α is continuously produced and degraded in the proteasome, while under hypoxic conditions, the HIF-α protein stabilizes and translocates from the cytoplasm to the nucleus, where it binds to HIF-1β and forms an activated HIF dimer complex [7]. The activated HIF complex then associates with hypoxia-response elements (HREs) in the regulatory regions of target genes and binds transcriptional coactivators to induce the transcription of more than 100 genes implicated in many biological processes, such as cellular metabolism, inflammation, angiogenesis, proliferation and apoptosis [8,9]. Studies have reported an important role for HIF-α in liver IRI. Activation of HIF-α alleviates hepatic IRI by inhibiting FAS-mediated apoptosis [10]. Moreover, hypoxia activates the HIF-α pathway in liver epithelial cells and regulates mitochondrial metabolism by transcribing PDK1 and maintaining ATP levels in mitochondria [11]. As shown in our recent study, preconditional activation of the HIF-1 pathway by a HIF-1α agonist (FG-4592) protects the donor liver obtained after cardiac death (DCD) from warm ischemia and cold-storage injury [12]. However, the molecular mechanisms underlying the protective effect of HIF-1α on hepatic IRI are not fully understood.

Extracellular adenosine is another signaling molecule that has been recognized as playing an important role in the cellular adaptation to hypoxia [13]. Upon cell injury or death initiated by IRI, precursor nucleotides, such as ATP, ADP, or AMP, are released from injured cells and subsequently act as a ‘danger signal’ to propagate inflammation and immunoreactions [14]. Meanwhile, hypoxia and ischemia also trigger the transcriptional induction of CD39 (ectonucleoside triphosphate diphosphohydrolase 1), which converts ATP/ADP to AMP, and CD73 (ecto-5ʹ nucleotidase), which converts AMP to adenosine. These enzymes ultimately hydrolyze ATP to adenosine, contributing to adapting the cells to anoxic environments and attenuating inflammation [14]. Extracellular adenosine signaling is known to have a role in protecting the liver from IRI and triggers cellular responses through interactions with four distinct adenosine receptors (ARs), A1, A2A, A2B, and A3, but researchers have not clearly determined which receptor mediates hepatoprotective effects [15]. Among them, A2B adenosine receptor (A2BAR) is the most adenosine-insensitive receptor that is activated only under pathological conditions, such as hypoxic or ischemic conditions and other forms of cellular stress [16]. A2BAR is reported to have a wide distribution in the heart, liver, lungs, kidneys and blood vessels and plays a role in the tissue adaptation to conditions of hypoxia, inflammation, or ischemia [17]. Importantly, inactivation of A2BAR via genetic deletion or pharmacological inhibition significantly aggravates liver injury caused by IRI [16,18]. Additionally, hepatocellular-specific A2BAR signaling contributes important protective actions against hepatic IRI by attenuating NF-κB activation and subsequent inflammation [18].

Several studies have found that HIF-1α induces the expression of A2BAR and its subsequent activation in endothelial cells [19] and dendritic cells [20], acute lung injury [21], liver cancer [22] and breast cancer [23] under hypoxic stress. However, the association between HIF-1α and A2BAR signaling during hepatic IRI has not yet been investigated. Therefore, a reasonable speculation is that HIF-1α plays a role in hepatic IRI by regulating A2BAR signaling. In the present study, we performed in vitro and in vivo experiments to identify the role of HIF-1α in hepatic IRI and to explore the related regulatory mechanism with the purpose of identifying novel therapies for hepatic IRI.

## Materials and methods

### Animals

Male Wistar rats weighing 250–300 g were obtained from the Animal Experiment Center of Wuhan University. Rats were maintained under standard animal care conditions with free access to water and a standard chow diet. The experimental protocols were reviewed and approved by the Animal Experiment Committee of Wuhan University, and all the rats were handled according to the National Institutes of Health Guide for Care and Use of Laboratory Animals.

### Model establishment and experimental design

A total liver IRI model was established as described previously [24]. Briefly, rats were anaesthetized with pentobarbital sodium (40 mg/kg) via intraperitoneal injection. The hepatic hilum was exposed by a midline laparotomy and was clamped with a microvascular clip to induce ischemia. After 30 minutes of occlusion, reperfusion was achieved for 6 h by loosening the clip. No rats died during reperfusion. Rats were randomly divided into four groups (6 rats per group) as follows:

#### Sham group

rats were treated with laparotomy and mobilization of the hepatic hilum but without vascular occlusion;

#### IRI group

rats were treated with vehicle (dimethyl sulfoxide, 0.1% DMSO, Sigma-Aldrich, USA) via intraperitoneal injection 6 h before laparotomy with ischemia;

#### FG-4592 group

rats were treated with a HIF-1α agonist, FG-4592 (6 mg/kg, Selleckchem, USA), via intraperitoneal injection 6 h before laparotomy with ischemia; and

#### 2ME2 group

rats were treated with a HIF-1α inhibitor, 2ME2 (6 mg/kg, Selleckchem, USA), via intraperitoneal injection 6 h before laparotomy with ischemia.

The doses and injection times of FG-4592 and 2ME2 were based on our previous study [12] and the study by Hoppe G *et al* [25], respectively. All groups of rats were humanely sacrificed at 6 h after reperfusion, and blood and liver tissue samples were collected and stored until further experiments.

### Serum biochemical analysis

Serum levels of alanine aminotransferase (ALT) and aspartate aminotransferase (AST) were detected using an automatic biochemistry analyzer at the clinical laboratory of the Central South Hospital of Wuhan University.

### Oxidative stress analysis

Oxidative stress in tissue and cells was assessed by measuring the formation of malondialdehyde (MDA) and superoxide dismutase (SOD) using a colorimetric assay kit (Nanjing Jiancheng Bioengineering Institute, Nanjing, China) according to the manufacturer’s protocols.

### Cytokine analysis

Serum tumor necrosis factor α (TNF-α) and interleukin-6 (IL-6) levels were measured using ELISA kits purchased from Shanghai Enzyme-linked Biotechnology Co., Ltd. (Shanghai, China) according to the manufacturer’s instructions.

### Myeloperoxidase activity analysis

The activity of myeloperoxidase (MPO) in liver tissue was assayed according to the instructions provided with a commercial kit (Nanjing Jiancheng Bioengineering Institute, Nanjing, China).

### Histopathological analysis and TUNEL staining

Liver tissues were fixed with formalin and embedded in paraffin. Tissue sections (5 µm) were stained with hematoxylin and eosin to evaluate the extent of hepatic tissue damage according to Suzuki’s criteria [26]. The score ranged from 0 to 12, depending on the degree of sinusoidal congestion (score: 0–4), cellular vacuolization (score: 0–4) and necrosis (score: 0–4). Apoptosis was detected using a terminal deoxynucleotidyl transferase dUTP nick end labeling (TUNEL) assay (Roche Diagnostics, Indianapolis, IN, USA) according to the manufacturer’s instructions. The number of total hepatocytes and TUNEL-positive cells was counted in three randomly chosen fields (× 100 magnification) from each liver section under a fluorescence microscope. The percentage of apoptosis (TUNEL-positive cells/total number of hepatocytes ×100%) in each image was analyzed with Image-Pro Plus 6.0 software (Media Cybernetics, Rockville, MD, USA).

### Quantitative real-time polymerase chain reaction (qRT-PCR)

Total RNA was isolated and purified from liver tissue and BRL-3A cells using TRIzol reagent (Invitrogen Inc., Grand Island, NY, USA). RNA was reverse transcribed to complementary DNAs (cDNAs) using the Thermo Scientific Revert Aid First Strand cDNA Synthesis Kit (Gene Copoeia, USA). Real-time PCR assays were performed using SYBR Green Master Mix on an ABI Prism 7000 platform (Applied Biosystems, Foster City, California, USA). Quantitative PCR results were obtained with the 2^(−ΔΔCt)^ method using β-actin as the reference transcript. All primers are listed in Table 1.

**Table 1.** Primers used for qRT-PCR

Gene		Primer sequence (5ʹ-3ʹ)
HIF1α	Forward	TTTCAAGCAGCAGGAATTGGAACG
	Reverse	TCTGCTCCATTCCATCCTGTTCAC
A1AR	Forward	GCCTACATTGGCATCGAGGT
	Reverse	GGTCTGTGGCCCAATGTTGA
A2AAR	Forward	TTAGCCCTCCCAGGGACATT
	Reverse	AGGCGACTTCGAAACTAGCG
A2BAR	Forward	GGAACACGAGCAAGAGGGAT
	Reverse	TGGTGGCACGGTCTTTACTG
A3AR	Forward	AGCCAACAATACCACGACGA
	Reverse	ATGTTGCCCACTACAGCACA
β-actin	Forward	TGCTATGTTGCCCTAGACTTCG
	Reverse	GTTGGCATAGGTCTTTACGG

### Western blotting

Western blotting was performed as previously described [12]. The following rabbit polyclonal antibodies were used: anti-HIF-1α (1:1000, Proteintech, Manchester, UK), anti-A2BAR (1:500, Bioss Company, Beijing, China), anti-Bcl-2 (1:1000, Proteintech, Manchester, UK), anti-Bax (1:1000, Proteintech, Manchester, UK), and anti-β-actin (1:5000, Proteintech, Manchester, UK). The protein bands were revealed using an enhanced chemiluminescence (ECL) reagent. Protein expression was measured by performing a densitometry analysis using ImageJ software (NIH, Bethesda, MD, USA).

### Culture and processing of cells

BRL-3A cells, a normal rat liver cell line, were purchased from the Cell Resource Center of the Chinese Academy of Sciences (Shanghai, China). Cells were cultured and maintained in Dulbecco’s modified Eagle’s medium (DMEM) (high sugar) supplemented with 5% fetal bovine serum (FBS) and 1% penicillin/streptomycin at 37°C with 21% O_2_, 5% CO_2_ levels and saturated humidity. When the cells had grown to 90% confluence, the supernatant was discarded. Adherent cells were washed twice with PBS, and serum-free DMEM (low sugar) was added. Then, the cells were placed in a saturated trigas incubator at 37°C in an atmosphere of 94% N2, 5% CO_2_ and 1% O2 for 12 h to elicit hypoxia. Afterward, the supernatant was discarded, fresh total medium was added, and the cells were reoxygenated in a CO_2_ incubator for 6 h. Cells were pretreated with different concentrations of FG-4592 (10, 20, 30, and 40 μM) for 6 h before the hypoxia treatment to determine whether the protective effects of HIF-1α activation under hypoxic conditions depended on A2BAR expression.

### RNA interference

Small interfering RNAs (siRNAs) corresponding to HIF-1α (Gene Bank mRNA accession number NM_024359.1) and A2BAR (Gene Bank mRNA accession number NM_017161.1) were designed and synthesized by ViewSolid Biotechnology (Beijing, China). Cells were grown to 50% confluence and transfected with the HIF-1α/A2BAR siRNA or a negative control siRNA (NC siRNA) for 24 h using LipoJet transfection reagent (Signagen Laboratories, USA). The cells were incubated with fresh reagents for another 24 h prior to 12 h of hypoxia exposure. We constructed three HIF1α-siRNAs, three A2BAR-siRNAs, and one NC-siRNA, and these siRNA target sequences were as follows: HIF1α-siRNA-1: 5ʹ-GAGCUCCCAUCUUGAUAAATT-3ʹ (forward) and 5ʹ-UUUAUCAAGAUGGGAGCUCTT-3ʹ (reverse); HIF1α-siRNA-2: 5ʹ-GGGCCGUUCAAUUUAUGAATT-3ʹ (forward) and 5ʹ-UUCAUAAAUUGAACGGCCCTT’ (reverse); HIF1α-siRNA-3: 5ʹ-GCCUCUUCGACAAGCUUAATT-3ʹ (forward) and 5ʹ-UUAAGCUUGUCGAAGAGGCTT-3ʹ (reverse); A2BAR-siRNA-1: 5ʹ-CCCAUGAGCUACAUGGUUUTT-3ʹ (forward) and 5ʹ-CCCAUGAGCUACAUGGUUUTT-3ʹ (reverse);

A2BAR-siRNA-2: 5ʹ-CCCUAGCUGUGUAUUAUAUTT-3ʹ (forward) and 5ʹ-AUAUAAUACACAGCUAGGGTT-3ʹ (reverse); A2BAR-siRNA-3: 5ʹCCCAUGAGCUACAUGGUUUTT-3ʹ (forward) and 5ʹ-AAACCAUGUAGCUCAUGGGTT-3ʹ (reverse); NC-siRNA: 5ʹ-AAACCAUGUAGCUCAUGGGTT-3ʹ (forward) and 5ʹ-ACGUGACACGUUCGGAGAATT’ (reverse); A2BAR-siRNA forward (+) 5ʹ-CCCAUGAG CUACAUGGUUUTT-3ʹ, reverse (–) 5ʹ-AAACCAUGUAGCUCAUG GGTT-3ʹ; and NC-siRNA forward (+) 5ʹ-UUCUCCGAACGUGUCACGU TT-3ʹ, reverse (–) 5ʹ- ACGUGACACGUUCGGAGAATT −3ʹ.

### Flow Cytometry

The Annexin V-FITC Apoptosis Detection Kit (BD Pharmingen, UK) was used to identify DNA fragmentation and plasma membrane alterations occurring during hypoxia-induced apoptosis in BLR-3A cells. Apoptotic cells were stained with Annexin V-FITC and propidium iodide (PI) and analyzed using a FACSCalibur flow cytometer (Becton Dickinson, CA, USA) as previously described. The data were analyzed using CellQuest software (Becton Dickinson, CA, USA). Intracellular reactive oxygen species (ROS) levels were measured using the 2′,7′-dichlorodihydrofluorescein diacetate (DCFH-DA) probe (Sigma) by flow cytometry. Briefly, cells were harvested after treatment and washed twice with PBS, incubated with DCFH-DA (1 μM) in serum-free medium at 37°C for 20 minutes, washed twice with PBS and analyzed using a flow cytometer. The data were processed using FlowJo software (Tree Star, San Carlos, CA, USA).

## Statistical analysis

Statistical analyses were performed using SPSS 21.0 (SPSS, Chicago, IL, USA). All data are presented as the means ± SD. Comparisons between two groups were performed using the unpaired t-test, and comparisons among multiple groups were analyzed using one-way ANOVA followed by a Tukey post hoc test. P < 0.05 was considered statistically significant.

## Results

### HIF-1α expression is upregulated during liver IR or H/R injury

**Figure 1.**
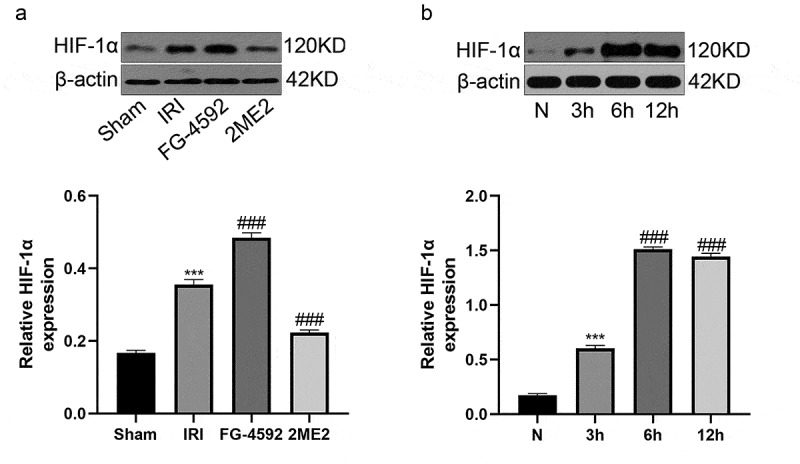
HIF-1α expression is upregulated after liver IR or H/R injury. (a) Western blotting analysis of HIF-1α expression and the band density ratio relative to β-actin in liver tissues of each group. (b) Western blotting analysis of HIF-1α expression and the band density ratio relative to β-actin in BRL-3A cells in different reoxygenation times (0, 3 h,6 h, and 12 h). ***P < 0.001 versus the sham or N group; ^###^ P < 0.001 versus the IRI or 3 h group

We first monitored the HIF-1α expression level in the liver of rats subjected to IRI to examine whether this protein plays a role in liver IRI. Western blot analyses revealed increased expression of the HIF-1α protein in the IRI group compared with the sham group. Meanwhile, compared with the IRI group, the expression level of HIF-1α in the FG-4592 group was significantly upregulated, while it was markedly downregulated in the 2ME2 group (Figure 1a). We constructed an H/R model with a time gradient of reoxygenation (3 h, 6 h and 12 h) to further explore the effect of HIF-1α at the cellular level. As shown in the Western blot analysis, HIF-1α expression was gradually increased in a time-dependent manner and peaked at 6 h after reoxygenation in BRL-3A cells (Figure 1b). According to the results, we chose 6 h of reoxygenation as the parameter for further experiments. These results suggested that the expression level of HIF-1α was increased following liver IRI in vivo and in vitro, and it may play an important role in regulating liver IRI.

### HIF-1α reduces liver damage induced by IR in rats

**Figure 2.**
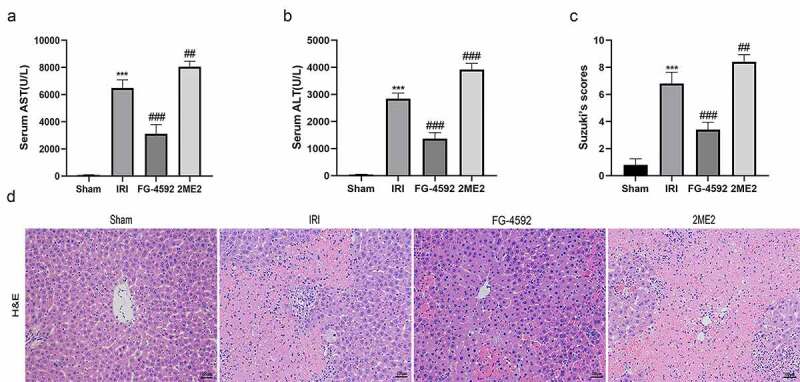
HIF-1α reduces hepatic injury induced by IR in rats. Serum AST (a) and ALT (b) levels of each group. (c) The degree of injury is scored based on Suzuki’s scores. (d) Representative images for the histological analysis of the HE-stained liver tissues at 200× magnification. Scale bars: 100 μm. All data are shown as mean ± SEM (n = 6 per group). ***P < 0.001 versus the sham group; ^##^P < 0.01 and ^###^P < 0.001 versus the IRI group

We detected the serum levels of the aminotransferases ALT and AST in each group to investigate whether HIF-1α exerts protective effects on liver IRI. Significant higher levels of ALT and AST were detected in the IRI group than in the sham group, and these levels were markedly lower in the FG-4592 group than in the IRI group. Moreover, considerably higher ALT and AST levels were observed in the 2ME2 group than in the IRI group (Figure 2a and Figure 2b). Histopathological changes in the liver after reperfusion for 6 h were also analyzed, and the degree of injury was graded by recording Suzuki histopathological scores. Consistent with the ALT and AST alterations, liver damage was more serious in rats from the IRI group than that in the sham group, as evidenced by extensive sinusoidal congestion, hepatocyte necrosis, vacuolar degeneration, inflammatory cell infiltration, and higher Suzuki’s scores. Furthermore, rats from the FG-4592 group showed markedly decreased hepatic injury and Suzuki’s scores, while the degree of hepatic injury was significantly increased in rats from the 2ME2 group compared to those from the IRI group (Figure 2c and Figure 2d). Collectively, HIF-1α exerted a protective effect on liver IRI.

### HIF-1α ameliorates inflammatory responses during hepatic IRI in rats

**Figure 3.**
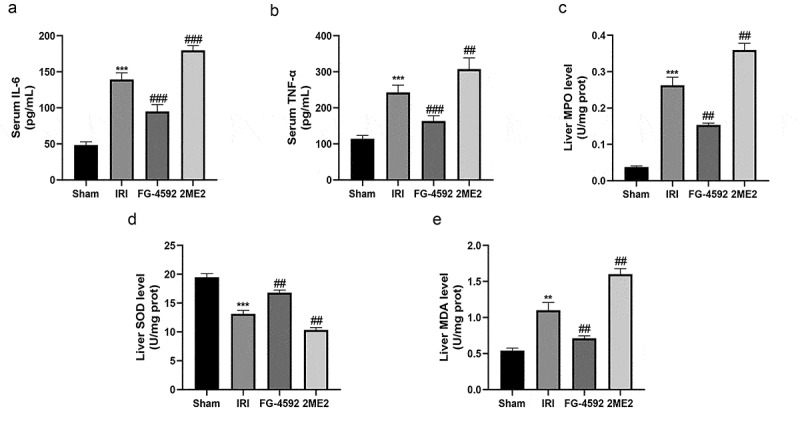
HIF-1α alleviates hepatic inflammatory responses and oxidative stress during hepatic IRI in rats. The levels of the serum IL-6 (a), TNF-α (b) and liver MPO (c), SOD (d) and MDA (e) in each group. All data are shown as mean ± SEM (n = 6 per group). **P < 0.01 and ***P < 0.001 versus the sham group; ^##^P < 0.01 and ^###^P < 0.001 versus the IRI group

Severe aseptic inflammation is an important feature of liver IRI. TNF-α and IL-6 are important proinflammatory cytokines that are produced during liver IRI and cause inflammatory damage to the liver. Furthermore, the MPO assay in liver tissue indirectly reflects the degree of neutrophil infiltration. Hence, we tested the serum TNF-α and IL-6 levels and the MPO content in liver tissues to explore the effect of HIF-1α on the systemic inflammatory response in rats during liver IRI. As depicted in Figure 3a-c, compared with the sham group, the level of hepatic infiltration of neutrophils (MPO) and the serum levels of TNF-α and IL-6 were remarkably increased in the IRI group. Significantly lower levels of IL-6, TNF-α and MPO were detected in the FG-4592 group and significantly higher levels were detected in the 2ME2 group than in the IRI group. Based on these results, HIF-1α significantly alleviated inflammatory responses during liver IRI.

### HIF-1α alleviates oxidative stress-induced damage during hepatic IRI in rats

During liver IRI, mitochondrial damage in hepatocytes leads to increased MDA level and decreased SOD level, which serve as important biomarkers for the assessment of oxidative damage. As shown in Figure 3d and Figure 3e, compared to the sham group, SOD level in hepatic tissues from the IRI group were significantly reduced, and the MDA concentration was significantly increased. A lower level of SOD and higher MDA concentration were observed in the 2ME2 group than in the IRI group. Conversely, compared to the IRI group, the SOD level was significantly higher and the MDA concentration was significantly lower in the FG-4592 group. Therefore, HIF-1α alleviated oxidative stress-induced damage during liver IRI.

### HIF-1α protects the rat liver from IRI-induced cell apoptosis

**Figure 4.**
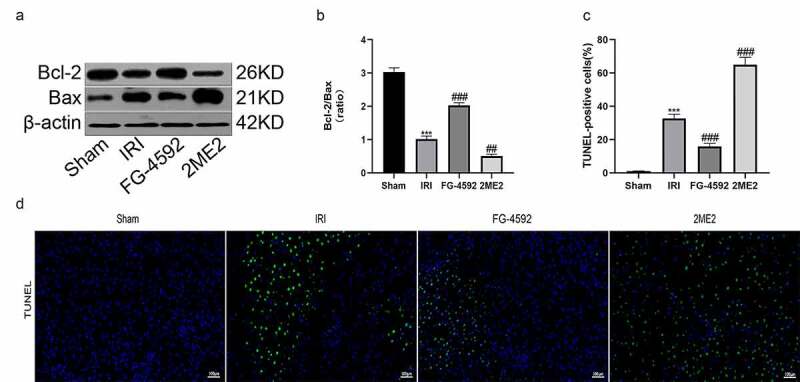
HIF-1α protects the liver from IRI-induced cell apoptosis in rats. (a) Western blot analysis of Bcl-2 and Bax expression in liver tissues of each group (β-actin is used as a loading control). (b) The fold change analysis of Bcl-2/Bax. (c) TUNEL-positive cells were quantified from 5 randomly selected fields at 200× magnification. (d) Representative images of TUNEL staining in liver sections of each group. Scale bars: 100 μm. All data are shown as mean ± SEM (n = 6 per group). ***P < 0.001 versus the sham group; ^##^P < 0.01 and ^###^P < 0.001 versus the IRI group

Apoptosis is one of the pathways of cell death that occurs in the process of liver IRI. We performed western blotting to detect changes in the levels of apoptosis-related proteins (Bcl-2 and Bax) and to examine whether HIF-1α exerted a protective effect on liver IRI-induced cell damage. The Bcl-2/Bax ratio was lower in the IRI group than in the sham group. Compared to the IRI group, the Bcl-2/Bax ratio was significantly increased in the FG-4592 group and decreased significantly in the 2ME2 group (Figure 4a and Figure 4b). Moreover, liver tissue sections were subjected to TUNEL staining. The results revealed a higher level of apoptosis in the IRI group than in the sham group, and the level of apoptosis in the FG-4592 group was lower than that in the IRI group. However, a significant increase in the number of apoptotic cells was observed in the 2ME2 group compared with the IRI group (Figure 4c and Figure 4d). Collectively, these results indicated that HIF-1α protected the liver from IRI-induced cell death.

### Knockdown of HIF-1α aggravates H/R-induced oxidative stress and hepatocyte apoptosis in vitro

**Figure 5.**
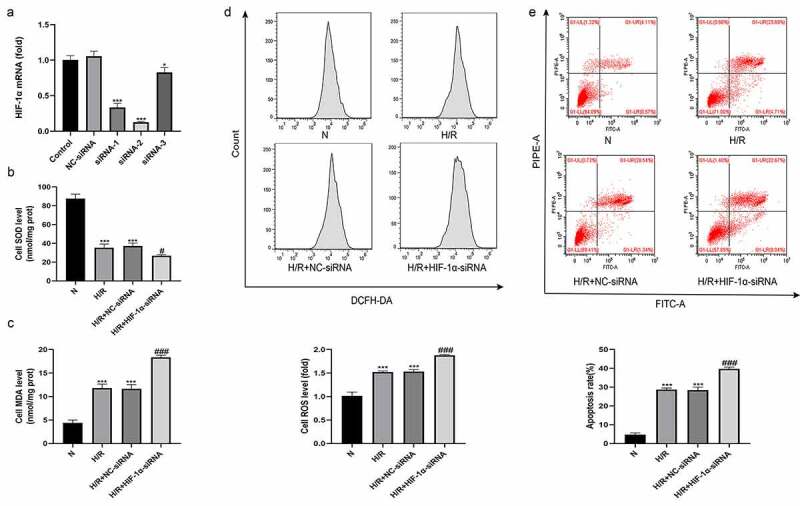
Knockdown of HIF-1α aggravates hepatic injury induced by H/R in vitro. (a) The effect of HIF-1α-siRNA transfection on HIF-1α expression was examined by qRT-PCR. The levels of the SOD (b) and MDA (c) in BRL-3A cells of each group. (d) Intracellular ROS generation of BRL-3A cells assayed and quantified analysis by flow cytometry. (e) Apoptosis of BRL-3A cells assayed and quantitative analysis of the percentage of apoptotic cells by flow cytometry. All data are shown as mean ± SEM (n = 6 per group). *P < 0.05 and ***P < 0.001 versus the NC-siRNA or N group; ^#^P < 0.05 and ^###^P < 0.001 versus the H/R+ NC-siRNA group

A hypoxia/reoxygenation (H/R) model was established using BLR-3A cells to explore the effect of changes in HIF-1α function on hepatocytes. We also constructed plasmids containing three HIF-1α-siRNAs and one NC-siRNA for loss-of-function experiments, and the expression level of HIF-1α was detected using qRT-PCR. As shown in Figure 5a, HIF-1α-siRNA-2 was better at silencing HIF-1α expression than the other two plasmids. Therefore, HIF-1α-siRNA-2 was used to knockdown HIF-1α expression. Four treatment groups were established as follows: normal (N), H/R, H/R+ NC-siRNA, and H/R+ HIF-1α-siRNA. We first used flow cytometry to detect oxidative stress-induced injury by measuring ROS levels in each treatment group. Simultaneously, we measured the MDA and SOD contents in each group of cells. As shown in Figure 5b-d, compared with the N group, the levels of ROS and MDA in the cells of the H/R group were significantly increased, and the SOD content was significantly reduced. No significant differences in the levels of ROS, MDA and SOD were observed between the H/R+ NC-siRNA group and the H/R group. However, cells from the H/R+ HIF-1α-siRNA group had higher levels of ROS and MDA and lower level of SOD than those from the H/R+ NC-siRNA group. We also used flow cytometry to detect the apoptosis rate of cells from each group. Compared with the N group, the apoptosis level of cells from the H/R group was significantly higher. The apoptosis level of cells from the H/R+ NC-siRNA group was not significantly different from that of the H/R group. However, cells from the H/R+ HIF-1α-siRNA group had a higher apoptosis rate than those from the H/R+ NC-siRNA group (Figure 5e). Based on these results, HIF-1α effectively protected BLR-3A cells from H/R-induced damage.

### HIF-1α is associated with A2BAR expression in hepatic IR or H/R injury

**Figure 6.**
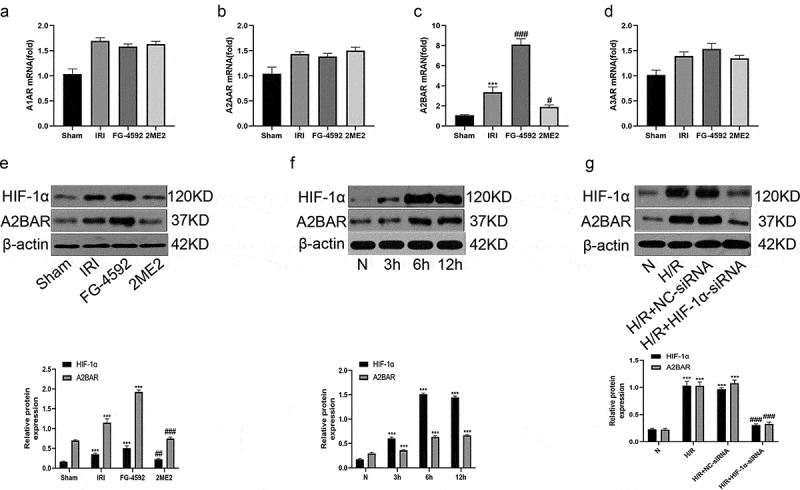
HIF-1α is associated with A2BAR in hepatic IR or H/R injury. (a-d) The expression levels of adenosine receptors (A1, A2A, A2B and A3) in liver tissue of each group by qRT-PCR. (e) Western blot bands and quantitative analysis of HIF-1α and A2BAR expression of liver tissues in each group (β-actin is used as a loading control). (f) Western blot bands and quantitative analysis of HIF-1α and A2BAR expression in BRL-3A cells in different reoxygenation times (0, 3 h,6 h, and 12 h) (β-actin is used as a loading control). (g) Western blot bands and quantitative analysis of HIF-1α and A2BAR expression in cells of each group (β-actin is used as a loading control). All data are shown as mean ± SEM (n = 6 per group). ***P < 0.001 versus the sham or N group; ^#^P < 0.05 and ^###^P < 0.01 versus the IRI or H/R+ NC-siRNA group

The obvious protective effects of HIF-1α on IRI-induced liver damage prompted us to investigate the mechanisms underlying these effects. Four adenosine receptors (ADORA1, 2A, 2B and A3) have been identified, but researchers have not clearly determined which receptor mediates hepatoprotective effects. In in vivo experiments, we first detected the mRNA expression levels of adenosine receptors A1AR, A2AAR, A2BAR, and A3AR in the liver tissues of rats from each group using qRT-PCR. As illustrated in Figure 6a-d, no significant differences in the mRNA expression levels of A1AR, A2AR and A3AR were observed among all groups. However, the expression level of the A2BAR mRNA in the IRI group was significantly higher than that in the sham group. Compared with the IRI group, the expression level of the A2BAR mRNA was significantly increased in the FG-4592 group and significantly decreased in the 2ME2 group. The findings were further confirmed by the Western blot analysis. As shown in Figure 6e-g, consistent with the trend of HIF-1α levels, A2BAR protein levels were significantly increased in the IRI group compared with the sham group, while this increase was markedly inhibited by the 2ME2 pretreatment. Moreover, rats pretreated with FG-4592 exhibited a marked increase in the liver level of the A2BAR protein. With similar results obtained from our in vitro experiments, Western blot analysis showed that the expression of A2BAR protein was significantly increased in a time-dependent manner and peaked at 12 h after reoxygenation in the BRL-3A cell H/R model, and this increase was significantly inhibited by the HIF-1α-siRNA. Collectively, a correlation was observed between HIF-1α and A2BAR signaling during liver IRI.

### Overexpression of HIF-1α alleviates H/R-induced oxidative stress and hepatocyte apoptosis in vitro

**Figure 7.**
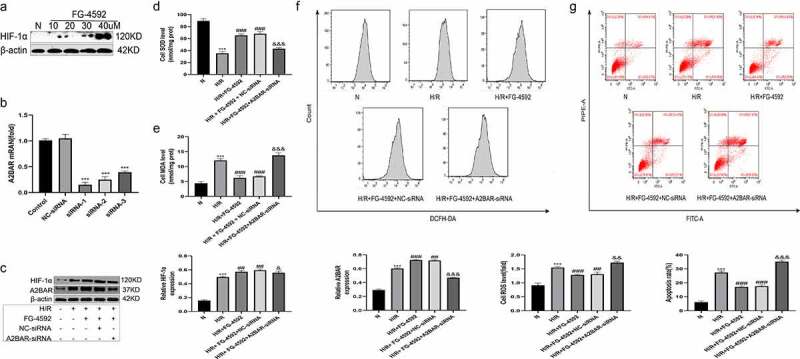
Overexpression of HIF-1α alleviates hepatic injury induced by H/R in vitro and blocking the A2BAR abrogates the protective effects. (a) Western blot analysis of HIF-1α expression in BRL-3A cells in different concentrations of FG-4592. (b) The effect of A2BAR-siRNA transfection on HIF-1α expression was examined by qRT-PCR. (c) Western blot bands and quantitative analysis of HIF-1α and A2BAR expression in cells of each group (β-actin is used as a loading control). The levels of the SOD (d) and MDA (e) in BRL-3A cells of each group. (f) Intracellular ROS generation of BRL-3A cells assayed and quantified analysis by flow cytometry. (g) Apoptosis of BRL-3A cells assayed and quantitative analysis of the percentage of apoptotic cells by flow cytometry. All data are shown as mean ± SEM (n = 6 per group). ***P < 0.001 versus the NC-siRNA or N group; ^##^P < 0.05 and ^###^P < 0.01 versus the H/R group; ^&^P < 0.05, ^&&^P < 0.01 and ^&&&^P < 0.001 versus the H/R+ FG-4592+ NC-siRNA group

We added the selective HIF-1α agonist FG-4592 to the cell culture medium 6 h before H/R treatment to induce the expression of HIF-1α in BLR-3A cells and to further confirm the protective effect of HIF-1α on hepatocytes. We treated BLR-3A cells with different concentrations of FG-4592 (10, 20, 30, and 40 µM) followed by H/R treatment and then subjected these cells to Western blot analysis to determine the proper dosage. As shown in Figure 7a, the expression of the HIF-1α protein in cells increased in a dose-dependent manner. Hence, we employed 40 µM FG-4592 to treat BLR-3A cells. The experimental groups were as follows: normal (N), H/R, and H/R+ FG-4592. Cells from the H/R group had higher levels of ROS and MDA, lower levels of SOD, and higher cell apoptosis rates than those from the N group. However, compared with the H/R group, cells pretreated with FG-4592 exhibited significantly reduced levels of ROS and MDA, an increased SOD level, and the inhibition of cell apoptosis (Figure 7d-g). These results further confirmed that HIF-1α effectively protected BLR-3A cells from H/R-induced damage.

### A2BAR blockade abrogates the protective effects of HIF-1α on H/R injury to BRL-3A cells

Based on the results obtained, we speculated that HIF-1α exerted protective effects on liver IRI by regulating A2BAR. We constructed plasmids containing three A2BAR-siRNA (A2BAR-siRNA1, A2BAR-siRNA2, and A2BAR-siRNA3) to knockdown A2BAR expression in BLR-3A cells and further verify our speculation; the expression level of A2BAR was detected using qRT-PCR. As shown in Figure 7b, A2BAR-siRNA1 most effectively inhibited H/R-induced A2BAR expression. Thus, we used A2BAR-siRNA1 in subsequent experiments. We also added the selective HIF-1α agonist FG-4592 (40 µM) to the cell culture medium at 24 h after cell transfection, followed by H/R treatment 6 h later. Five treatment groups were established as follows: normal (N), H/R, H/R+ FG-4592, H/R+ FG-4592+ NC-siRNA, and H/R+ FG-4592+ A2BAR-siRNA. As shown in Figure 7c, the expression of the A2BAR protein in the H/R+ FG-4592+ A2BAR-siRNA group was significantly lower than that in the H/R+ FG-4592 group, indicating that A2BAR expression was effectively suppressed. No significant differences in the levels of ROS, MDA, SOD, and apoptosis were observed between the H/R+ FG-4592 group and the H/R + FG-4592 + NC-siRNA group. However, the ROS levels, MDA levels, and apoptosis rates were higher, and the SOD level was lower in the H/R+ FG-4592+ A2BAR-siRNA group than in the H/R+ FG-4592+ NC-siRNA group (Figure 7d-g). Therefore, the HIF-1α overexpression-mediated protective effects on BRL-3A cells were abolished by siRNA-A2BAR, thus confirming that HIF-1α exhibited protective effects on liver IRI by upregulating A2BAR signaling.

## Discussion

IRI is an inevitable complication during liver surgery that seriously affects the prognosis of patients. The pathological processes of hepatic IRI are complex and involve liver tissue damage, oxidative stress, inflammation, and hepatocyte apoptosis, among others [5]. Although many studies have examined hepatic IRI, its mechanism has not been clearly explained. Currently, an ideal drug or treatment method is unavailable for hepatic IRI. Thus, an urgent need is to investigate the molecular pathophysiological mechanisms responsible for hepatic IRI to develop new targets and drugs for the treatment of hepatic IRI. In the present study, we observed a significant increase in the HIF-1α level in the rat liver subjected to IRI, as well as in BRL-3A cells treated with H/R, and overexpression of HIF-1α exerted a protective effect on hepatic IRI or H/R insult by reducing liver damage, inhibiting inflammation and hepatocyte apoptosis, and alleviating oxidative stress. In contrast, inhibition of HIF-1α expression exacerbated hepatic injury induced by IR or H/R. In addition, the potential mechanisms of HIF-1α overexpression-mediated protection against liver IRI were associated with the upregulation of A2BAR signaling. Here, treatments targeting the HIF-1α/A2BAR signaling axis may be a promising therapeutic approach to ameliorate hepatic IRI.

HIF-1α is the main transcription factor in cells that is activated in response to hypoxia/ischemia and induces the expression of numerous target genes to activate protective physiological responses and promote cell survival under hypoxic conditions [6,8]. HIF-1α exhibits a variety of functions in diverse pathological conditions. Hypoxia-induced HIF-1α plays a role in the regulation of neutrophil bactericidal activity and inhibits neutrophil apoptosis [27]. Studies have shown that hypoxia directly contributes to the progression of liver fibrosis and that HIF-1α represents an important regulator of hepatic stellate cell (HSC) activation by modulating autophagy [28]. Moreover, activation of HIF-1α under hypoxic stress regulates the transcription of genes that participate in key pathways involved in carcinogenesis, such as angiogenesis, dedifferentiation, and invasion [29]. Importantly, HIF-1α activation has been shown to induce a variety of protective mechanisms against IRI in multiple organs. HIF-1α inhibits neuronal apoptosis following cerebral IRI in rats partially by upregulating EPO expression [30]. Overexpressed HIF-1α reduces oxidative stress and inhibits the opening of the mPTP, thus achieving protection against myocardial IRI [31]. In addition, HIF-1α attenuates pulmonary vascular dysfunction during lung IRI in rats through effects on the iNOS/NO pathway [32]. Similarly, our data also clearly indicate that HIF-1α functions as a protective factor in hepatic IRI or H/R insult through its antioxidant, anti-inflammatory, and antiapoptotic effects.

During hepatic IRI, the redox balance is dysregulated, and the normal functions and integrity of tissues are damaged, resulting in the accumulation of ROS [33]. Increased ROS production is a major detrimental event that causes hepatocyte damage and even death, partly because ROS attack various critical biological lipids, particularly membrane phospholipids, leading to the formation of reactive aldehydes, such as MDA, to further aggravate injury [34]. SOD is an important antioxidant that catalyses the oxidation of reactive oxygen species into inactive substances and oxygen and protects cells against damage caused by IRI-induced ROS production [33]. Previous studies have shown that upregulated HIF-1α alleviates oxidative stress-related injury by reducing ROS production from the mitochondria and thus protects against IRI in the heart [35]. HIF-1α also attenuates oxidative stress-induced apoptosis by directly targeting mitochondria [36]. Our results similarly showed that HIF-1α overexpression increased SOD activity but reduced ROS levels and MDA concentrations in response to hepatic IRI or H/R insult, indicating that HIF-1α may alleviate oxidative stress-mediated injury during hepatic IR or H/R.

Recently, researchers proposed that the excessive release of inflammatory factors plays a pivotal role in the pathogenesis of hepatic IRI [37]. TNF-α and IL-6 are considered important mediators of inflammation and result in aggravated liver damage after reperfusion by promoting the recruitment of neutrophils, which reflect the degree of hepatic IRI, and thus the inhibition of both cytokines can attenuate liver injury [38]. Accumulating evidence has suggested an important role for HIF-1α in executing the immune response, the activation of which has either anti-inflammatory or proinflammatory effects on multiple organ systems, depending on its pathophysiology context [39]. For instance, HIF-1α activation has been implicated in the proinflammatory response of myeloid cells during sepsis, and deletion of HIF-1α in myeloid cells afforded protection against LPS-induced mortality [40]. Similarly, HIF-1α activation exacerbates the T/HS-induced gut- and lung-derived inflammatory response and regulates cytokine expression (TNF-α and IL-1) in the ileal mucosa and lung [41]. Conversely, HIF-1α exerts a robust anti-inflammatory effect on the hypoxic gastrointestinal mucosa and epithelium [42]. The results of the present study indicated that HIF-1α overexpression reduced the release of inflammatory factors (TNF-α and IL-6) and neutrophil infiltration (as indicated by MPO staining) during hepatic IRI. Our findings suggested that HIF-1α alleviated the inflammatory responses induced by hepatic IRI.

Apoptosis is a process of programmed cell death resulting from various stress stimuli, such as hypoxia, oxidative stress and DNA damage, where the Bcl-2 protein family plays an important role, especially Bcl-2 and Bax. The Bcl-2/Bax ratio determines the cell fate in response to an apoptotic stimulus [43,44]. During liver IRI, a large number of hepatic parenchymal cells undergo apoptotic death after an ischemic insult, and the inhibition of apoptosis protects against reperfusion injury [45]. Additionally, the expression of HIF-1α significantly correlates with apoptosis and proapoptotic factors, such as caspase-3, Fas, and Fas ligand [46]. Previous studies have indicated that HIF-1α inhibits neuronal apoptosis following cerebral ischemia in rats by upregulating EPO [30]. Consistent with previous findings, our present study revealed that HIF-1α overexpression resulted in increased levels of Bcl-2, reduced levels of Bax and numbers of TUNEL-positive cells in livers subjected to IRI, and decreased the proportion of apoptotic BLR-3A cells exposed to the H/R insult, suggesting that HIF-1α alleviated the degree of apoptosis resulting from hepatic IR or H/R injury.

While exploring the possible downstream target genes mediating these protective effects of HIF-1α on hepatic IRI, we found that the expression level of A2BAR was significantly increased in the rat liver subjected to IRI and in BRL-3A cells exposed to H/R insult, which showed the same trends as HIF-1α. However, the expression levels of A1AR, A2AAR and A3AR were not remarkably changed. This finding was consistent with previous studies reporting that A2BAR transcription is dramatically induced by hypoxia, and protected the liver from IRI in mice with a single knockout of each adenosine receptor [18]. Moreover, overexpression of HIF-1α increased A2BAR expression, and the expression level of A2BAR was decreased when HIF-1α was downregulated. Based on these results, HIF-1α is potentially associated with the pathway during hepatic IR or H/R injury. A2BAR is expressed on a wide variety of cell types and has been shown to exert crucial anti-inflammatory and tissue-protective effects on many models of ischemia and inflammation [17]. Previous studies have confirmed that A2BAR plays an important role in protection against IRI in different organs, such as the heart [47], liver [18], intestinal tract [48], and kidney [49]. In hepatic IRI activation of hepatocellular-specific A2BAR protects the liver from IRI by attenuating NF-κB activation and subsequent liver cell inflammation [18]. A2BAR ^−/-^ mice exhibited significantly higher levels of tissue injury than their corresponding littermate controls during liver IRI [15]. Moreover, A2BAR plays a major role in the anti-inflammatory actions of sevoflurane during liver IRI [50]. Importantly, emerging evidence has indicated that HIF-1α, a master hypoxia-induced transcriptional regulator, mediates the expression and activation of A2BAR in multiple cells and tissues [19–23]. Furthermore, the A2BAR promoter has been reported to contain a functional HIF-1α binding site, and HIF-1α may regulate the expression of A2BAR by binding to the HRE in the A2BAR promoter region [19]. We conducted loss-of-function experiments in vitro to further validate whether the protective effects of HIF-1α on hepatic IRI depended on downstream A2BAR signaling and found that upregulated HIF-1α increased the expression level of A2BAR and protected BLR-3A cells from H/R injury by alleviating cell apoptosis and oxidative stress. However, A2BAR silencing with an siRNA abolished the protective effects of HIF-1α overexpression. Together, the aforementioned results suggest that HIF-1α may exert its protective effects on hepatic IRI by upregulating A2BAR signaling.

However, our study has a few limitations. First, the accuracy in this experiment must be improved by increasing the number of animal models. Second, the role of HIF-1α in protecting against hepatic IRI could be further illustrated using genetic tools with specific HIF-1α overexpression or knockdown. However, these tools are not currently available. Third, this study preliminarily discussed the relationship between HIF-1α and A2BAR during hepatic IRI, although further studies are needed to determine the specific regulatory mechanism and contributions of HIF-1α to A2BAR expression. We will attempt to address these questions in our future studies.

## Conclusions

In summary, our study is the first to show that HIF-1α attenuates liver necrosis, the inflammatory response, oxidative stress and apoptosis during hepatic IRI by upregulating A2BAR signaling, thereby preventing liver damage. This study provides a novel theoretical basis for the treatment of hepatic IRI.

## Article Highlights

1. HIF-1α expression is upregulated during hepatic IR or H/R injury.
2. Overexpression of HIF-1α exerts a protective role during hepatic IRI or H/R insult.
3. Inhibition of HIF-1α expression exacerbates hepatic injury induced by IR or H/R.
4. HIF-1α protects the liver against IRI through upregulation of the A2B adenosine receptor.
